# Effects of surgical trauma and intraoperative blood loss on tumour progression

**DOI:** 10.3389/fonc.2024.1412367

**Published:** 2024-06-07

**Authors:** Xiaoqin Jin, Han Han, Qilian Liang

**Affiliations:** Oncology Center, Affiliated Hospital of Guangdong Medical University, Zhanjiang, China

**Keywords:** haemorrhage, surgical trauma, tumour growth, immunosuppression, tumour recurrence

## Abstract

Surgery is the primary treatment of choice for tumours, and improves prognosis, prolongs survival and is potentially curative. Previous studies have described the effects of anaesthesia and changes in the neuroendocrine, circulatory and sympathetic nervous systems on postoperative cancer progression. There is growing evidence that intraoperative blood loss is an independent prognostic factor for tumour recurrence, postoperative inflammation is a predictor of cancer prognosis, and immunosuppressive status correlates with the degree of surgical damage. This paper outlines the potential mechanisms by which blood loss, surgical trauma and postoperative immunosuppressive status contribute to tumour growth and recurrence by reducing intraoperative haemorrhage and perioperative immunotherapy, thereby reducing tumour growth and recurrence, and improving long-term prognosis.

## 1 Introduction

Surgical resection is the most promising therapeutic tool for malignant tumours. Several clinical studies have shown that postoperative metastatic recurrence rate of many tumours, such as gastric cancer, breast cancer, hepatocellular carcinoma and colorectal cancer, is high and closely related to surgery (1–3). Surgery sometimes results in excessive intraoperative blood loss (IBL) and the need for perioperative allogeneic blood transfusion. There is controversy as to whether IBL or perioperative transfusion has an adverse prognostic impact on patients with malignant tumours. There are a lot of clinical data showing the association between poor prognosis and significant IBL, but the mechanism of the adverse effects of perioperative blood transfusion and its prognostic impact is beyond the scope of this review. The inflammatory response and immunosuppression caused by surgical trauma may promote tumour cell growth, migration and recurrence. Therefore, we review the potential mechanisms of tumour growth and recurrence triggered by surgical trauma, IBL and postoperative immunosuppression, and discuss the current methods used to reduce tissue trauma and IBL and perioperative immunotherapy.

## 2 Impact of IBL on tumour growth and recurrence

Transfusion-associated immunosuppression and immune escape have long been considered to be potentially associated with tumour recurrence and metastasis, and IBL is reported to be associated with prognosis in many malignancies (4–7). A recent study by Nakanishi et al. (8) questioned the relevance of blood transfusion to tumour recurrence. At the same time, an increasing body of research indicates that trauma-related inflammation, immunosuppression, bleeding and tumour metastasis are all independently correlated. Nakanishi et al. suggested that the adverse prognostic impact of IBL may be due to three factors: antitumour immunosuppression caused by IBL; postoperative complications; and formation of microscopic metastatic foci of residual intraoperative tumour cells as a result of IBL. Firstly, many studies have shown that the adverse effects of excess IBL are mainly caused by antitumour immunosuppression through impaired immunity to cancer cells and loss of plasma components, and that the immunotrophic status of patients plays an important role in tumour immunity (9–12). However, these studies did not establish the mechanisms involved. Secondly, excessive IBL may lead to the development of postoperative complications, which may adversely affect prognosis (11, 13). Finally, Kamei et al. (14) reported that excessive IBL was an independent risk factor for peritoneal recurrence after radical gastrectomy. They concluded that blood loss was significantly associated with peritoneal recurrence and that blood loss into the peritoneal cavity may promote spillage of residual tumour cells during surgery.

It has been demonstrated that IBL is an independent predictor of overall survival following radical gastrectomy in patients with stage I–III gastric cancer (10, 14). **Table 1** summarises studies of the prognostic impact of IBL. The adverse prognostic impact of IBL in patients with gastric cancer was first reported by Dhar et al. (9) in 2000. Their analysis of 152 patients with transmural (T2N0–T3N2) gastric cancer concluded that IBL >500 mL was an independent prognostic factor for postoperative survival. Citing Bruns et al. (19), they hypothesised that IBL reduces the body’s immunity and its ability to fight cancer cells. However, Dhar et al. (9) did not provide information on perioperative blood transfusion, which is an important prognostic confounder. Kamei et al. (14) first reported the relationship between IBL and pattern of recurrence in 2009. IBL ≥475 mL was positively correlated with peritoneal metastasis in 146 patients who underwent radical gastrectomy for advanced gastric cancer. They stated that IBL is another independent risk factor for peritoneal recurrence after radical resection for advanced gastric cancer, rather than for haematological or local recurrence. Liang et al. (10) also reported that IBL ≥200 mL was an independent prognostic factor in 845 patients who underwent radical gastrectomy. Even after excluding patients who underwent blood transfusion, their study showed that IBL, but not blood transfusion, was an independent prognostic factor after radical gastrectomy for gastric cancer. Mizuno et al. (11) reported that IBL ≥400 mL was a significant prognostic factor for survival and tumour recurrence in patients with stage II/III gastric cancer. They excluded patients receiving blood transfusion, to eliminate confounding factors caused by transfusion-related adverse reactions. Ito et al. (12) reported that IBL >330 mL was associated with poor prognosis in patients with stage II/III gastric cancer, and they also excluded patients who received blood transfusion. There are still few studies on the correlation between IBL,which excludes blood transfusion, and long-term prognosis of patients with gastric cancer (10–12). IBL is closely associated with blood transfusion; therefore, the prognostic significance of IBL may be masked by the adverse effects of transfusion. The above three studies (10–12) excluded this confounding factor, suggesting that IBL *per se* has a detrimental impact on long-term prognosis in patients with gastric cancer. Hiroshi et al. (15) reported that IBL >400 mL was associated with poor prognosis of Borrmann type IV gastric cancer, and intraoperative bleeding control had a positive impact on survival in patients undergoing radical resection for gastric cancer. A meta-analysis (20) of 4653 patients reported that greater IBL after gastrectomy in patients with gastric cancer resulted in decreased long-term survival and recurrence-free survival.

**Table 1:** Studies on the prognostic impact of IBL.

Study	Period	Sample size	Selected group	Amount of IBL	Patients received BTF	Adverse effect of IBL on OS▴	Adverse effect of IBL on RFS ▴
Dhar et al (9) 2000	1979-1989	152	Gastric Cancer T2N0-T3N2	>500ml	Not specified	Yes	HR1.779, p=0.0352
Kamei et al (14) 2009	1992-2003	146	Curative Gastrectomy	>475ml	Included 13%	Yes (Peritoneal recurrence)	HR1.0019(95%CI)(1.0001-1.0041), p=0.0352
Liang et al (10) 2013	2003-2007	845	Stage I-III Gastric Cancer	>200ml	Included 25%	Yes	HR1.590(95% CI)(1.140-2.217), p=0.006
Akira et al (11) 2016	1999-2015	203	Stage II-III Gastric Cancer	>400ml	Included 0%	HR1.72(95% CI)(1.03-2.87), p=0.04	Yes
Ito et al (12) 2018	2010-2014	1013	Stage II-III Gastric Cancer	>330ml	Not specified	Yes	HR1.45(95% CI)(1.01-2.09), p=0.0420
Hiroshi et al (15) 2020	1995-2016	2789	Borrmann Type IV Gastric Cancer	>400ml	Not specified	HR1.64(95% CI)(1.01-2.67), p=0.045	Yes
Hiroshi et al (6) 2020	2007-2012	76	Stage II/III Pancreatic Cancer	>1000ml	Not specified	HR2.391(95% CI)(1.166-4.903), p=0.017	Yes
Hiroshi et al (16) 2021	2000-2019	1597	Stage II/III Colorectal Cancer	>200ml	Not specified	HR2.730(95% CI)(1.647-4.524), p <0.001	HR1.713(95% CI)(1.348-2.178), p <0.001
Suk-won et al (4) 2023	2010-2021	142	hepatic resection for HCC	>700ml	Included 25.3%	*HR8.390(95% CI)(1.044–67.408), p=0.045	HR2.325(95% CI)(1.202–4.497, p = 0.012
Hayato et al (7)2023	2011-2019	198	Locally Advanced Esophageal Cancer	>850ml	Not specified	HR2.091(95% CI)(1.120-3.906), p=0.021	HR1.811(95% CI)(1.030-3.184), p=0.039
Rahul et al (17)2017	1995-2016	611	Laparoscopic hepatectomy for HCC	>250ml	Included 5.4%	OR2.48(95% CI 1.524-4.031) p = 0.0001	Yes
Xiaocui et al (18) 2021	2001-2019	192	Laparoscopic hepatectomy for HCC	>250ml	Included 20%	OR0.5(95% CI)(0.3–0.9), p<0.02	OR0.5(95% CI)(0.3–0.8), p<0.02

▴Multifactorial analysis of the adverse effect of IBF on OS and RFS. *Univariate Analysis of the adverse effect of IBF on OS.

IBL during surgery has also been shown to be an independent predictor of long-term survival and tumour recurrence after radical surgery for other gastrointestinal tumours, including hepatocellular, colorectal, oesophageal and pancreatic carcinomas. These authors concluded that successful radical resection and limited blood loss contribute to improved survival. In hepatocellular carcinoma, Suk-Won et al. (4) found by multivariate analysis that excessive IBL was an independent prognostic factor for tumour recurrence after hepatectomy. A meta-analysis of 1540 colorectal cancer patients showed that lower IBL resulted in better overall survival (OS) and disease-free survival, as well as a lower rate of postoperative complications (5). A multicentre retrospective study found 89.3% OS and 63.4% disease-free survival at 5 years postoperatively in patients with high IBL after radical surgery for colorectal cancer (16). Multifactorial analysis has shown that IBL is an independent risk factor for OS in patients with pancreatic invasive ductal adenocarcinoma treated with pancreatectomy (6). Minimising IBL intraoperatively is important. It has been found that positive para-abdominal aortic lymph nodes and the status of the R0 and R1 margins after pancreatic adenocarcinoma resection are important independent prognostic factors (21, 22). In patients with locally advanced oesophageal cancer, Hayato et al. reported that severe IBL may be a useful predictor of postoperative recurrence and OS (7). Although IBL seems to be an important and sensitive indicator, none of the aforementioned studies on its impact on survival, prognosis and recurrence of gastrointestinal tumours has elucidated its possible underlying mechanisms. In addition, there are discrepancies in the literature regarding whether IBL is an independent prognostic factor, particularly with regard to tumour growth and recurrence. IBL is an independent deleterious factor that negatively affects patient prognosis, promotes tumour growth and recurrence, and should be considered in the context of other factors that influence tumour recurrence (e.g., surgical scope and timeline, laparoscopic versus open surgery, tissue trauma, patient comorbidities, cardiac function, psychological stress, anaesthetic management and surgical approach) (22, 23).

Numerous studies have shown that laparoscopic surgery reduces blood loss and transfusion compared with open surgery (24–27). However, the evidence for improved immune and endocrine function after lumpectomy is not yet sufficient. The fact that laparoscopic surgery does not yet offer significant advantages may be related to the complexity of its operative procedures, especially for abdominal tumour surgery. Although randomised clinical trials have demonstrated significantly lower levels of tumour markers after laparoscopy compared with open surgery, there were no significant differences in terms of 3-year survival and recurrence rates (24). Laparoscopic colorectal cancer surgery usually involves more abdominal organs, prolongs the operating time, and may trigger endocrine and immune stress responses in the same way as open surgery (28). Furthermore, it has been shown that IBL is likely to be underestimated in laparoscopic hepatectomy (29). Rahul et al. (17) also reported that IBL ≥250 mL during laparoscopic hepatectomy may adversely affect postoperative outcomes. In a retrospective study of 192 patients with hepatocellular carcinoma who underwent laparoscopic surgery, Xiaocui et al. (18) found that increased intraoperative bleeding was an independent predictor of tumour recurrence. Thus, all these studies clearly indicate that excessive IBL is an important factor in the process of tumour metastasis.

## 3 Effect of surgical trauma on tumour growth and recurrence

**Figure 1:**
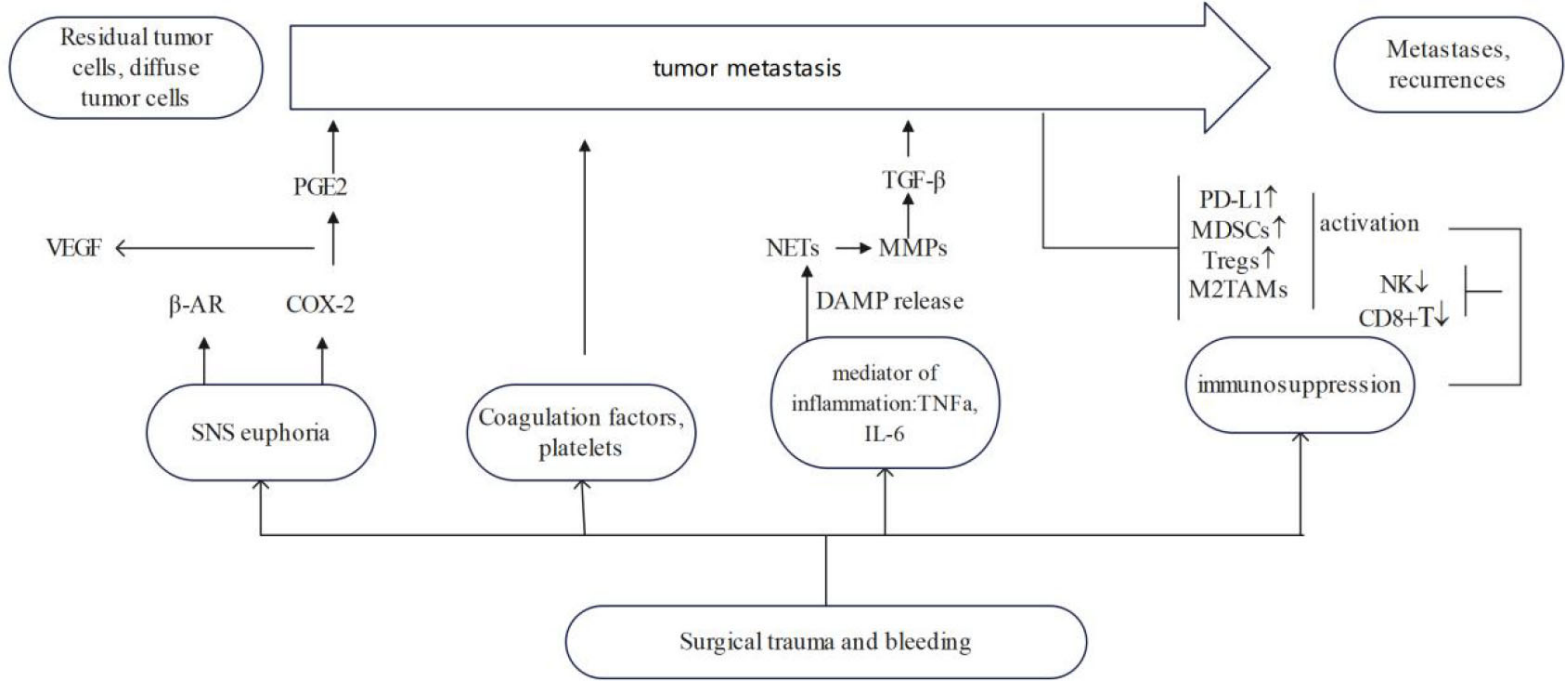
Effect of surgical trauma on tumor growth and recurrence. ↓ means decline, ↑ means ascension.

Surgical trauma with haemorrhage is often accompanied by an early excessive inflammatory response and significant suppression of immune function (**Figure 1**). Following surgical resection of the tumour, the sympathetic nervous system activates and promotes the release of catecholamines, with concomitant haemovolumic insufficiency and vasoconstriction mediated by sympathetic reflexes and a surge of catecholamines, leading to reduced blood flow and inadequate oxygen delivery to peripheral tissues and organs. Subsequently, this hypoxic state may lead to decreased cellular ATP levels, cellular calcium homeostatic balance, as well as accumulation of lactic acid and oxygen free radicals and anaerobic cellular metabolism. This may stimulate the release of inflammatory mediators such as cytokines and prostaglandin E2 (PGE2). Suppression of various immune functions is evident immediately after haemorrhage and persists for a long time despite administration of volume resuscitation. Increased release of inflammatory mediators is associated with significant suppression of the immune response and increased susceptibility to infection after haemorrhage, affecting various cell types such as macrophages and lymphocytes, ultimately leading to systemic immunosuppression (19). In addition to this, an additional perioperative risk factor that is often overlooked is psychosocial stressors, which can also trigger a variety of physiologic responses affecting metastasis. Patients experience anxiety, stress, and depression from the onset of a cancer diagnosis, which translates into activation of the SNS and the hypothalamic-pituitary-adrenal (HPA) axis (30), as well as the consequent release of stress hormones. Relevant literature has reviewed the impact of stress processes and stress management interventions on immune variables (e.g., cellular immune function, inflammation) that may or may not change in direct response to cancer or its treatment (31).

### 3.1 Surgical trauma induces an early hyperinflammatory response

Surgical trauma and IBL lead to sympathetic nervous system activation and promote catecholamine release, hypovolemia, vasoconstriction and tissue hypoxia, with an increased demand for anaerobic metabolism, leading to reduced ATP levels. The reduction in cellular ATP is associated with significant inhibition of splenocyte proliferation and lymphocyte factor production [i.e., interleukin (IL)-2 and tumour necrosis factor (TNF)-α] (32). Tissue hypoxia and enhanced anaerobic metabolism also lead to the accumulation of lactic acid and oxygen free radicals in the body. This lactate, as well as that produced by tumour cells, has been shown to have a key role in cell signalling and can lead to cellular dysfunction, altered macrophage reactivity and inhibition of antigen presentation, by inducing expression of vascular endothelial growth factor and M2-like polarisation of tumour-associated macrophages (33). Hypoxia also promotes genomic instability, leading to a variety of genetic changes that facilitate the formation of a more aggressive phenotype in residual tumour cells (34). Finally, reduced ATP levels also disrupt cellular calcium homeostasis. It has been shown that dysregulation of cytosolic calcium homeostasis may lead to cellular immunosuppression after haemorrhage by impairing macrophage antigen-presenting function and splenocyte function (35). The significant reduction of cellular ATP after surgical-trauma-induced early excessive inflammatory response, haemorrhage and local hypoxia may be responsible for early postoperative immunosuppression.

Surgical trauma directly induces the release of catecholamines, which are an important neuroendocrine response to tissue injury and its associated inflammatory response, injury perception and pain (36). There is growing evidence that catecholamines also exert immunosuppressive effects, promoting proliferation, migration and angiogenesis, as well as accelerating tumour growth and metastasis (37). Catecholamines can induce metastatic effects in tumour cells and the tumour microenvironment by stimulating β-adrenoceptors (βARs), which can lead to cancer recurrence, for instance, in mammary tumours, activation of β-adrenoceptors was linked to accelerated tumor growth (38), PGE2 is also a key mediator in the induction of tumour metastasis (39, 40). Most immune cells express adrenergic receptors, mainly β2 receptors, on their surface. Adrenoceptors density varies among cell types and natural killer (NK) cells have the highest density (36, 41). The acute stress response may inhibit NK cell activity by releasing catecholamines through the adrenal glands and activating β1- and β2-ARs, which in turn diminishes the resistance of NK cells to tumour metastasis and may lead to immunosuppression (36). Loza et al. (41) concluded that catecholamine activation of βARs in Th1 and Th2 cells, rather than Th1/Th2 transition, leads to immunosuppression. Results from animal model studies confirm that stress-induced neuroendocrine activation has a negligible effect on primary tumour growth but induces a 30-fold increase in metastasis to distant tissues, including lymph nodes and lungs. These stress responses are mediated by βARs, which increases the infiltration of CD11b^+^ F4/80^+^ macrophages into the primary tumour parenchyma, thereby inducing M2 macrophage differentiation and prometastatic gene expression (42).

Catecholamines can increase the risk of cancer by creating an environment receptive to metastatic growth of diffuse tumour cells via βARs (43). Upregulation of proangiogenic factors such as vascular endothelial growth factor (VEGF) and neurotrophic factors via βARs leads to upregulation of matrix metalloproteinases, which in turn promotes tumour growth and facilitates tumour invasion into the extracellular matrix (ECM) (44). It has also been shown that catecholamines have stronger effects on the immune system than glucocorticoids, and that sympathetic nervous system activation inhibits the killing of tumour cells by NK cells (45). In addition to their haemodynamic effects, catecholamines have many other properties and regulate key processes in tumour growth and metastatic spread. A randomised controlled trial demonstrated that perioperative cyclooxygenase-2 (COX-2) and βAR blockers improved biomarkers of metastasis, immunity and inflammation in colorectal cancer (46). Further research is needed into potential strategies, such as βAR blockade, to attenuate the deleterious effects of perioperative catecholamines, inhibit tumour growth, halt metastatic spread and ultimately improve treatment outcomes.

The direct action of catecholamines and prostaglandins on tumour tissue has only recently been recognised. Catecholamine and prostaglandin receptors are expressed in a large number of human cancers (47), and activation of these receptors promotes tumour metastasis through a variety of molecular mechanisms: pro-tumour cell proliferation, adhesion, migration, ECM invasion, apoptosis and shedding of apoptosis resistance, as well as secretion of proangiogenic cytokines such as VEGF. These mechanisms are essential for metastatic spread and growth of tumour cells, and therefore, blocking them may inhibit metastasis (48–50). The indirect effects of catecholamines and prostaglandins are mediated through a variety of mechanisms: suppression of perioperative antitumour metastatic immunity, changes in the protumourigenic properties of the microenvironment of residual tumour cells, and the stimulatory effects of lymphatic-mediated tumour cell spread (51–54).

### 3.2 Surgical-trauma-induced immunosuppression

#### 3.2.1 Cytokines

Surgical trauma and blood loss lead to the release of inflammatory cytokines [e.g., IL-10 and transforming growth factor (TGF)-β] and PGE2. Firstly, surgically induced stress response activates upregulation of COX-2, which directly reinforces tumour cell proliferation and metastatic invasion (46). The COX-2/PGE2 pathway is a classical proinflammatory pathway that is involved in various functions such as tumourigenesis, progression and metastasis (55). In addition, sympathetic nervous system activation can indirectly lead to the release of several protumour factors, including matrix metalloproteinase-9 (MMP-9), VEGF, TGF-β, IL-6 and IL-8, triggering a series of inflammatory responses to stimulate tumour recurrence and metastasis (48, 56). Secondly, surgical stress leads to increased PGE2 synthesis, which further inhibits macrophage and lymphocyte function (57), and it has been demonstrated that PGE2 is involved in the process of tumourigenesis, including tumour neovascularisation (58, 59). Finally, the rapid inflammatory reaction following surgical damage makes it easier for tumour cells to be captured at distant locations, which encourages metastasis. Proinflammatory cytokines such as IL-1 and TNF-α stimulate the adhesion of circulating cancer cells. In the inflammatory environment following surgery, dormant cancer cells can exhibit growth patterns and elude immunosurveillance (60). However, it has been demonstrated that perioperative anti-inflammatory medication reduces tumour growth and restores the immune response in this setting (61).

#### 3.2.2 Release of damage-associated molecular pattern during surgery induces neutrophil extracellular trap formation

Damage-associated molecular patterns (DAMPs) are key molecular ligands that trigger the inflammatory immune response after surgical injury, and neutrophils are rapidly recruited to the site of injury by DAMPs within a few minutes of surgery and induce the formation of neutrophil extracellular traps (NETs) (62). Neutrophils and the NETs that they form play an important role in cancer initiation, progression and metastasis (63). NETs not only awaken dormant tumour cells in mice during inflammation (64), but also carry circulating tumour cells (CTCs) to evade killing by immune cells, inducing the colonisation of distal organs by CTCs (65). NETs can also locally release factors such as MMP-9 and VEGF (63, 66). MMP-9 enhances the bioavailability of VEGF in the tumour microenvironment by hydrolysing the molecular binding domain of VEGF, thereby activating dormant tumour cells and triggering tumour metastasis (3, 64, 67). MMP-9 also accelerates the degradation of local ECM, leading to the release and activation of other tumour-promoting factors stored in the local ECM of NETs (63, 68). MMP-9 locally amplifies the protumourigenic effects of TGF-β, and activated TGF-β directly promotes tumour invasion and angiogenesis, and exerts extensive immunosuppression on the tumour microenvironment, further inducing tumour metastasis (69–71). In addition to neutrophils, inflammatory changes at the post-tumour resection site include the recruitment of macrophages and proinflammatory factors. Proinflammatory factors such as IL-1 and TNF-α enhance the adhesion of CTCs, which facilitates the capture of tumour cells at the outer margin site (60). Inflammation may also form premetastatic ecological niches by stripping the microcirculatory endothelium.

#### 3.2.3 Macrophages and NK cells

Tumour-associated macrophages (TAMs) are classified as M1 macrophages (classical activation pathway) and M2 macrophages (alternative activation pathway). M1 macrophages are mainly found in the inflammatory environment and can fight tumours by presenting antigens, while M2 macrophages are mainly found in the tumour microenvironment and can promote tumour proliferation, invasion and metastasis. Postoperatively produced PGE2 also promotes macrophage differentiation towards a tumour-promoting M2 phenotype (59). Recently, it has been shown that the transcription factor CCAAT/enhancer binding protein β inhibits the antitumour immune response by regulating β-adrenergic conversion of macrophages to the M2 phenotype (72). Predina et al. demonstrated in an experimental model of lung cancer that surgical resection significantly increased many tumour-promoting cytokines (VEGF, IL-1β, IL-6, IL-10, monocyte chemoattractant protein-1 and TGF-β) and decreased interferon (IFN)-γ (73). In addition, recurrent tumours have alternatively activated macrophages and T regulatory (Treg) cells, which prevent the recruitment of CD8^+^ T cells to the tumour, contributing to immunosuppression after surgery (73). Cancer cells also have the ability to take advantage of wound-healing macrophages with a phenotype similar to that of TAMs; a response that tumours can exploit to promote transendothelial migration and metastatic disease dissemination. In the breast cancer model(triple-negative and luminal subtype B breast cancer), the tumour utilises macrophages to adopt a phenotype similar to that produced by macrophages found in granulation tissue during the inflammatory response to wounding, which in the case of cancer, can promote transendothelial migration of tumour cells. More importantly, the expression of wound-like cytokine responses within tumours has been clinically associated with poor prognosis in a variety of cancers (74). This study demonstrated the ability of tumour cells to exploit the innate wound healing response of macrophages to promote metastatic spread (74). As mentioned above, macrophages are another important driver of tumour progression after surgical removal of tumours. Macrophage depletion may be a promising adjunct to surgical resection (75, 76). Tham et al. demonstrated that macrophages contribute to postoperative tumour recurrence and metastatic growth and that surgery increased tumour cell proliferation, which correlated with an increased density of macrophages within the tumour (75). Macrophages stimulated the formation of tumour spheres from tumour cells in postoperative mice but not control mice. Postoperative removal of macrophages by a diet containing the CSF-1R-specific kinase inhibitor Ki20227 significantly reduced tumour recurrence and eliminated enhanced metastatic growth (75).

Surgical intervention disrupts the body’s homeostatic balance, and plasma levels of the cytokine IFN-γ released by NK cells begin to decline 1 h after surgery (77). Impairment of T-cell proliferation and NK cell activity persists for ~2 weeks, peaking on postoperative days 5–7 (78). IFN-γ release from NK cells is suppressed for several months after surgery for a variety of malignancies (79, 80). Numerous studies have confirmed that surgically induced NK cell dysfunction leads to impaired clearance of metastases (79, 81–86), which may contribute to increased postoperative cancer recurrence and metastasis in preclinical animal models (54, 81, 82, 87–89) and in human patients (79, 82–86, 90). NK cell cytotoxicity is significantly and transiently reduced on day 7 after surgery and is increased in patients who undergo surgical resection of the colorectum and pancreas gradually recovered within 1 month after surgery (84, 91). Velasquez et al. found that postoperative NK cell cytotoxic activity was significantly reduced on postoperative day 5 in patients with primary bone cancer who underwent surgical treatment (92). In addition, surgical stress inhibits IFN-γ production by NK cells. Thus, perioperative treatments intended to improve NK cell function can be applied to lessen the spread and recurrence of cancer. Surgical trauma induces platelet activation. A protective fibrin and platelet coating around tumour cells prevents detection and attack by NK cells (89, 93) and mediates adhesion of tumour cells to endothelial cells and release of proangiogenic and mitogenic factors (93). Administration of low molecular weight heparin during surgery has been shown to block the binding of adhesion molecules (P-selectin) on platelets to tumour cells and to restore cytotoxicity of NK cells to prevent tumour metastasis (89).

### 3.3 Amplification of myeloid-derived suppressor cells, Treg cells and elevated postoperative PD-L1 expression

Surgical trauma and initiation of subsequent wound repair procedures can directly unrestrict the body’s immune system from tumours and trigger tumour growth (94). Surgery contributes to immune escape mainly by triggering postoperative downregulation of the adaptive immune response, such as a decrease in the number of cytotoxic T cells and NK cells in the postoperative period (84, 95) and an increase in the number of immunosuppressive cells (73). Among these, Treg cells are highly immunosuppressive CD4^+^ T cells that migrate to the site of inflammation and suppress effector lymphocytes after surgery, which is closely associated with tumour recurrence (96, 97). Treg cells secrete a variety of inhibitory cytokines to participate in tumour immunity, such as IL-10, IL-35 and TGF-β, and block metabolism through high expression of IL-2 (98). Myeloid-derived suppressor cells (MDSCs) accumulate in tumours and peripheral lymphoid organs and can suppress the immune effects of T cells, NK cells and dendritic cells, leading to tumour growth and metastasis (99–101). Tumour resection can induce the accumulation of MDSCs in specific distal organs such as the lungs, thereby forming a premetastatic niche conducive to tumour cell colonisation (102). MDSCs can suppress tumour immunity by mediating T-cell suppression through the induction of Treg cells and TGF-β secretion (103). In addition, surgery can lead to differentiation of TAMs towards the M2 type (59, 72), and an increase in M2 macrophages can lead to tumour proliferation, invasion and metastasis (104). At the same time, a series of progrowth factors triggered by surgery induce rapid expansion of Treg cells and MDSCs, while promoting wound healing (73). Thus, the perioperative period may represent an immune gap during which the extracellular environment is more favourable for residual tumour growth. It has been shown that surgical trauma increases PD-1 expression in T cells (105). Related experiments further indicated that PD-1/PD-L1 induce postoperative T-cell dysfunction in lung cancer patients, thereby increasing the risk of postoperative metastasis. The underlying mechanism may be associated with elevated expression of caspase-3 in T cells (106). In an *in vivo* study, administration of anti-PD-1 antibody significantly improved T-cell proliferation and partially reversed surgical-trauma-induced T-cell apoptosis (107).

## 4 Perspectives on possible methods to reduce adverse effects of IBL

**Figure 2:**
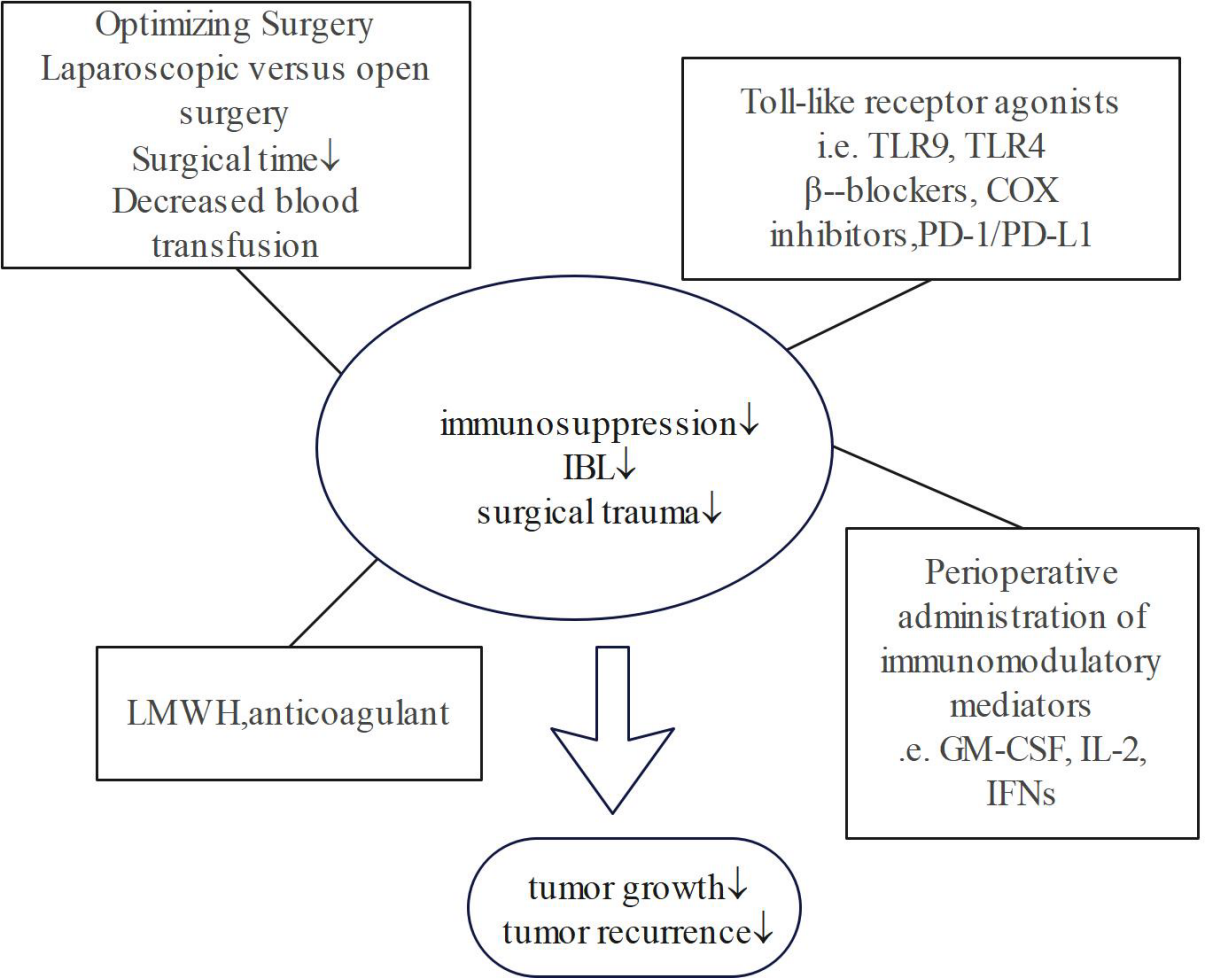
Summary of perioperative intervention methods. ↓ means decline.

Based on the recognition that IBL and surgical trauma are associated with tumour growth and recurrence, clinicians can adopt short-term and safe perioperative interventions to prevent tumour metastasis after surgery (**Figure 2**).

### 4.1 Optimising surgical procedures (perioperative interventions)

Optimising surgical procedures and reducing surgical trauma are the first steps in reducing IBL. Interventions for IBL require effective collaboration between the surgeon and the anaesthetic team. The perioperative immune response is influenced by the use of anaesthetics and analgesics, fluid resuscitation, blood transfusion, management of hypotensive episodes and hypothermia, pain, and the extent and duration of the procedure, and the severity of these factors correlates with immunosuppression (108). Numerous studies have demonstrated that laparoscopic surgery is more beneficial to postoperative recovery compared with open surgery, including reduced postoperative pain and analgesic use, as well as reduced blood loss and transfusion and shorter hospital stays (24–27, 109). Compared with patients after laparoscopic surgery, patients after caesarean section showed increased blood VEGF, matrix metalloproteinases (e.g., MMP-9), interleukins (e.g., IL-6), and TNF-α; cytokines that promote tumour growth and metastasis (110). A meta-analysis of surgical treatment for primary liver cancer showed that laparoscopic surgery caused less tissue damage and significantly less IBL compared with open surgery (27). In addition, recent studies have suggested that the pneumoperitoneum generated during laparoscopic surgery may have a procoagulant effect, thereby reducing intraoperative bleeding, however, the higher pressure is makes patients prone to complications such as hypercoagulability and deep vein thrombosis (111). Considering the potential immunosuppressive effects of blood transfusion, intraoperative blood salvage is an effective method to reduce the need for allogeneic blood transfusion (112). However, a comprehensive meta-analysis demonstrated that prognosis after the use of intraoperative blood salvage was not inferior to that of traditional intraoperative allogeneic transfusion, refuting the notion that there is a correlation between intraoperative blood salvage and an increased rate of metastasis or recurrence (113).

### 4.2 Immunotherapy

Because bleeding and intraoperative trauma cause severe immunosuppression, and perioperative interventions are often underutilised, safe and effective interventions that take advantage of this `window’ may be effective in preventing postoperative tumour recurrence and metastasis. Studies have shown that perioperative administration of immunomodulatory mediators, such as IL-2, IL-7, IFN and granulocyte–macrophage colony-stimulating factor, can reduce surgery-associated immunosuppression (114). A recent clinical trial showed that IL-7 restored lymphocytes and attenuated immunosuppression in patients with infectious shock (115). Another study has shown that IL-7 signalling in combination with immune checkpoint inhibitors enhances immunotherapy effectiveness and may reverse immunosuppression and reduce tumour growth (116). Immunostimulants can activate surgically induced immunosuppression. Preoperative use of immunostimulants Toll-like receptor (TLR)4 agonist GLA-SE and TLR9 agonist CpG-C was effective in blocking metastasis in a mouse model of liver metastasis from colon cancer (117). Clinical studies have shown that TLR4 activates T cells and dendritic cells and reduces cancer metastasis, without adverse effects (23). TLR9 agonists have been shown to activate NK cells and B cells (118), and to reduce tumour metastasis and growth in animal models (119). However, further clinical studies are required to assess their efficacy in the perioperative period and their safety. Similarly, administration of beta-blockers and selective COX-2 increased antitumour immunity and reduced the risk of tumour growth and metastasis in animal models (44). In recent years, immunotherapy has been rapidly developed and used for clinical treatment, and the development of immune checkpoint inhibitors has shown remarkable results in cancer immunotherapy. Immune checkpoint inhibitors such as anti-PD-1/PD-L1 antibodies and cytotoxic T-lymphocyte antigen-4 have been approved and used for perioperative treatment of a variety of malignant tumours. These act to promote immune-mediated clearance of tumour cells by targeting the blockade of common signalling pathways, thereby reactivating the antitumour immune response (120).

## 5 Conclusion

Surgical resection of the primary tumour remains the mainstay of treatment. Tissue trauma caused by surgery, intraoperative bleeding, postoperative paracrine and neuroendocrine responses, and postoperative immunosuppression may all have a role to play in tumour growth and recurrence. IBL has been shown to exacerbate the state of perioperative immunosuppression. Furthermore, excessive blood loss may function as a stand-alone predictor of tumour growth and recurrence. The aim of this review is to outline the potential mechanisms of how surgical trauma and successive perioperative inflammatory responses may affect tumour cell proliferation and growth. Recognition of the importance of the perioperative period may help surgeons develop strategies to reduce the incidence of IBL and postoperative immunosuppression, decrease tumour metastasis and improve tumour prognosis. In particular, for cancer types that necessitate significant surgical resection during removal of localised tumours, we emphasise surgical-trauma-induced immune dysfunction as a critical factor in decreasing postoperative cancer spread.

## Author contributions

XJ: Writing – original draft. HH: Data curation, Writing – review & editing. QL: Writing – review & editing.

## Glossary

**Table:**

IBL	Intraoperative blood loss
BTF	Blood transfusion
OS	Overall survival
DFS	disease-free survival
RFS	Relapse-free survival
HR	Hazard ratio
CI	Confidence interval
HCC	hepatocellular carcinoma
SNS	sympathetic nervous system
PGE-2	prostaglandin E2
IL-2	interleukin-2
IL-6	interleukin-6
IL-8	interleukin-8
TNF-α	tumor necrosis factor alpha
NK cells	nature kill cells
VEGF	vascular endothelial growth factor
βAR	β-adrenoceptors
COX-2	cyclooxygenase 2
ECM	extracellular matrix
MMP-9	matrix metalloproteinase-9
TGF-β	transforming growth factor beta
DAMPs	damage-associated molecular patterns
NETs	neutrophil extracellular traps
CTCs	Circulating tumor cells
TAMs	tumor-associated macrophages
C/EBPβ	CCAAT/enhancer binding protein β
IFN-γ	interferon
Tregs	regulatory T cells
MDSCs	myeloid-derived suppressor cells
PD-1	programmed death-1
PD-L1	programmed death ligand-1
LMWH	low molecular weight heparin
IBS	Intraoperative blood salvage
TLRs	Toll-like receptors
ICIs	immune checkpoint inhibitors
GM-CSF	granulocyte-macrophage colony-stimulating factor

## References

[1] Colleoni M Sun Z Price KN Karlsson P Forbes JF Thürlimann B. Annual hazard rates of recurrence for breast cancer during 24 years of follow-up: results from the international breast cancer study group trials I to V. J Clin Oncol. (2016) 34:927–35. doi: 10.1200/jco.2015.62.3504 PMC4933127 26786933

[2] Tsilimigras DI Pawlik TM. ASO author reflections: recurrence patterns and outcomes after resection of hepatocellular carcinoma within and beyond the barcelona clinic liver cancer criteria. Ann Surg Oncol. (2020) 27:2332–3. doi: 10.1245/s10434-020-08455-0 32297083

[3] Tohme S Yazdani HO Al-Khafaji AB Chidi AP Loughran P Mowen K. Neutrophil extracellular traps promote the development and progression of liver metastases after surgical stress. Cancer Res. (2016) 76:1367–80. doi: 10.1158/0008-5472.Can-15-1591 PMC4794393 26759232

[4] Suh SW Lee SE Choi YS. Influence of intraoperative blood loss on tumor recurrence after surgical resection in hepatocellular carcinoma. J Pers Med. (2023) 13. doi: 10.3390/jpm13071115 PMC10381288 37511728

[5] Li ZW Shu XP Wen ZL Liu F Liu XR Lv Q. Effect of intraoperative blood loss on postoperative complications and prognosis of patients with colorectal cancer: A meta−analysis. BioMed Rep. (2024) 20:22. doi: 10.3892/br.2023.1710 38169991 PMC10758914

[6] Tamagawa H Aoyama T Yamamoto N Kamiya M Murakawa M Atsumi Y. The impact of intraoperative blood loss on the survival of patients with stage II/III pancreatic cancer. In Vivo. (2020) 34:1469–74. doi: 10.21873/invivo.11931 PMC7279845 32354948

[7] Watanabe H Kano K Hashimoto I Tanabe M Onuma S Morita J. Intraoperative blood loss impacts recurrence and survival in patients with locally advanced esophageal cancer. Anticancer Res. (2023) 43:5173–9. doi: 10.21873/anticanres.16718 37909994

[8] Nakanishi K Kanda M Kodera Y. Long-lasting discussion: Adverse effects of intraoperative blood loss and allogeneic transfusion on prognosis of patients with gastric cancer. World J Gastroenterol. (2019) 25:2743–51. doi: 10.3748/wjg.v25.i22.2743 PMC6580348 31235997

[9] Dhar DK Kubota H Tachibana M Kotoh T Tabara H Watanabe R. Long-term survival of transmural advanced gastric carcinoma following curative resection: multivariate analysis of prognostic factors. World J Surg. (2000) 24:588–93. doi: 10.1007/s002689910099 10787082

[10] Liang YX Guo HH Deng JY Wang BG Ding XW Wang XN. Impact of intraoperative blood loss on survival after curative resection for gastric cancer. World J Gastroenterol. (2013) 19:5542–50. doi: 10.3748/wjg.v19.i33.5542 PMC3761109 24023499

[11] Mizuno A Kanda M Kobayashi D Tanaka C Iwata N Yamada S. Adverse effects of intraoperative blood loss on long-term outcomes after curative gastrectomy of patients with stage II/III gastric cancer. Dig Surg. (2016) 33:121–8. doi: 10.1159/000443219 26745751

[12] Ito Y Kanda M Ito S Mochizuki Y Teramoto H Ishigure K. Intraoperative blood loss is associated with shortened postoperative survival of patients with stage II/III gastric cancer: analysis of a multi-institutional dataset. World J Surg. (2019) 43:870–7. doi: 10.1007/s00268-018-4834-0 30377722

[13] Tokunaga M Tanizawa Y Bando E Kawamura T Terashima M. Poor survival rate in patients with postoperative intra-abdominal infectious complications following curative gastrectomy for gastric cancer. Ann Surg Oncol. (2013) 20:1575–83. doi: 10.1245/s10434-012-2720-9 23076557

[14] Kamei T Kitayama J Yamashita H Nagawa H. Intraoperative blood loss is a critical risk factor for peritoneal recurrence after curative resection of advanced gastric cancer. World J Surg. (2009) 33:1240–6. doi: 10.1007/s00268-009-9979-4 19308640

[15] Tamagawa H Aoyama T Kano K Numata M Atsumi Y Hara K. The impact of intraoperative blood loss on the long-term prognosis after curative resection for borrmann type IV gastric cancer: A retrospective multicenter study. Anticancer Res. (2020) 40:405–12. doi: 10.21873/anticanres.13967 31892594

[16] Tamagawa H Numata M Aoyama T Kazama K Atsumi Y Iguchi K. Impact of intraoperative blood loss on the survival of patients with stage II/III colorectal cancer: A multicenter retrospective study. In Vivo. (2021) 35:3483–8. doi: 10.21873/invivo.12649 PMC8627735 34697185

[17] Gupta R Fuks D Bourdeaux C Radkani P Nomi T Lamer C. Impact of intraoperative blood loss on the short-term outcomes of laparoscopic liver resection. Surg Endosc. (2017) 31:4451–7. doi: 10.1007/s00464-017-5496-y 28364154

[18] Lv X Zhang L Yu H Yu X. Laparoscopic hepatectomy for hepatocellular carcinoma: short- and long-term outcomes with blood loss. Transl Cancer Res. (2021) 10:4303–15. doi: 10.21037/tcr-21-463 PMC8797994 35116289

[19] Nagai S Fujii T Kodera Y Kanda M Sahin TT Kanzaki A. Impact of operative blood loss on survival in invasive ductal adenocarcinoma of the pancreas. Pancreas. (2011) 40:3–9. doi: 10.1097/MPA.0b013e3181f7147a 20881897

[20] Wen ZL Xiao DC Zhou X. Does intraoperative blood loss affect the short-term outcomes and prognosis of gastric cancer patients after gastrectomy? A meta-analysis. Front Surg. (2022) 9:924444. doi: 10.3389/fsurg.2022.924444 35774383 PMC9237360

[21] Arima K Hashimoto D Okabe H Inoue R Kaida T Higashi T. Intraoperative blood loss is not a predictor of prognosis for pancreatic cancer. Surg Today. (2016) 46:792–7. doi: 10.1007/s00595-015-1238-8 26302976

[22] Zhao B Huang X Lu H Zhang J Luo R Xu H. Intraoperative blood loss does not independently affect the survival outcome of gastric cancer patients who underwent curative resection. Clin Transl Oncol. (2019) 21:1197–206. doi: 10.1007/s12094-019-02046-6 30689183

[23] Matzner P Sorski L Shaashua L Elbaz E Lavon H Melamed R. Perioperative treatment with the new synthetic TLR-4 agonist GLA-SE reduces cancer metastasis without adverse effects. Int J Cancer. (2016) 138:1754–64. doi: 10.1002/ijc.29885 PMC4724303 26453448

[24] Lu YY Li YX He M Wang YL. Laparoscopic vs open surgery for gastric cancer: Assessing time, recovery, complications, and markers. World J Gastrointest Surg. (2024) 16:40–8. doi: 10.4240/wjgs.v16.i1.40 PMC10845286 38328321

[25] Bao D Hu Y Zhang C Jin Y Wang P Lin Y. Perioperative and short-term outcomes of laparoscopic liver resection for recurrent hepatocellular carcinoma: A retrospective study comparing open hepatectomy. Front Oncol. (2022) 12:956382. doi: 10.3389/fonc.2022.956382 36324570 PMC9618616

[26] Zhu Z Li L Xu J Ye W Zeng J Chen B. Laparoscopic versus open approach in gastrectomy for advanced gastric cancer: a systematic review. World J Surg Oncol. (2020) 18:126. doi: 10.1186/s12957-020-01888-7 32534587 PMC7293787

[27] Chen K Pan Y Hu GY Maher H Zheng XY Yan JF. Laparoscopic versus open major hepatectomy for hepatocellular carcinoma: A meta-analysis. Surg Laparosc Endosc Percutan Tech. (2018) 28:267–74. doi: 10.1097/sle.0000000000000567 30180140

[28] Sammour T Kahokehr A Zargar-Shoshtari K Hill AG. A prospective case-control study of the local and systemic cytokine response after laparoscopic versus open colonic surgery. J Surg Res. (2012) 173:278–85. doi: 10.1016/j.jss.2010.10.009 21195431

[29] Tomimaru Y Noguchi K Morita S Imamura H Iwazawa T Dono K. Is intraoperative blood loss underestimated in patients undergoing laparoscopic hepatectomy? World J Surg. (2018) 42:3685–91. doi: 10.1007/s00268-018-4655-1 29728731

[30] Thornton LM Andersen BL Blakely WP. The pain, depression, and fatigue symptom cluster in advanced breast cancer: covariation with the hypothalamic-pituitary-adrenal axis and the sympathetic nervous system. Health Psychol. (2010) 29:333–7. doi: 10.1037/a0018836 PMC2910549 20496988

[31] Antoni MH Dhabhar FS. The impact of psychosocial stress and stress management on immune responses in patients with cancer. Cancer. (2019) 125:1417–31. doi: 10.1002/cncr.31943 PMC6467795 30768779

[32] Meldrum DR Ayala A Wang P Ertel W Chaudry IH. Association between decreased splenic ATP levels and immunodepression: amelioration with ATP-MgCl2. Am J Physiol. (1991) 261:R351–7. doi: 10.1152/ajpregu.1991.261.2.R351 1877694

[33] Colegio OR Chu NQ Szabo AL Chu T Rhebergen AM Jairam V. Functional polarization of tumour-associated macrophages by tumour-derived lactic acid. Nature. (2014) 513:559–63. doi: 10.1038/nature13490 PMC4301845 25043024

[34] Reynolds TY Rockwell S Glazer PM. Genetic instability induced by the tumor microenvironment. Cancer Res. (1996) 56:5754–7. 8971187

[35] Chaudry IH Ayala A. Mechanism of increased susceptibility to infection following hemorrhage. Am J Surg. (1993) 165:59s–67s. doi: 10.1016/s0002-9610(05)81208-5 8439001

[36] Ben-Eliyahu S Shakhar G Page GG Stefanski V Shakhar K. Suppression of NK cell activity and of resistance to metastasis by stress: a role for adrenal catecholamines and beta-adrenoceptors. Neuroimmunomodulation. (2000) 8:154–64. doi: 10.1159/000054276 11124582

[37] Hartmann C Radermacher P Wepler M Nußbaum B. Non-hemodynamic effects of catecholamines. Shock. (2017) 48:390–400. doi: 10.1097/shk.0000000000000879 28915214

[38] Antoni MH Lutgendorf SK Cole SW Dhabhar FS Sephton SE McDonald PG. The influence of bio-behavioural factors on tumour biology: pathways and mechanisms. Nat Rev Cancer. (2006) 6:240–8. doi: 10.1038/nrc1820 PMC3146042 16498446

[39] Neeman E Ben-Eliyahu S. Surgery and stress promote cancer metastasis: new outlooks on perioperative mediating mechanisms and immune involvement. Brain Behav Immun. (2013) 30 Suppl:S32–40. doi: 10.1016/j.bbi.2012.03.006 PMC3423506 22504092

[40] Kim R. Effects of surgery and anesthetic choice on immunosuppression and cancer recurrence. J Transl Med. (2018) 16:8. doi: 10.1186/s12967-018-1389-7 29347949 PMC5774104

[41] Loza MJ Foster S Peters SP Penn RB. Beta-agonists modulate T-cell functions via direct actions on type 1 and type 2 cells. Blood. (2006) 107:2052–60. doi: 10.1182/blood-2005-08-3265 PMC1895713 16278302

[42] Sloan EK Priceman SJ Cox BF Yu S Pimentel MA Tangkanangnukul V. The sympathetic nervous system induces a metastatic switch in primary breast cancer. Cancer Res. (2010) 70:7042–52. doi: 10.1158/0008-5472.Can-10-0522 PMC2940980 20823155

[43] Hiller JG Perry NJ Poulogiannis G Riedel B Sloan EK. Perioperative events influence cancer recurrence risk after surgery. Nat Rev Clin Oncol. (2018) 15:205–18. doi: 10.1038/nrclinonc.2017.194 29283170

[44] Renz BW Takahashi R Tanaka T Macchini M Hayakawa Y Dantes Z. β2 adrenergic-neurotrophin feedforward loop promotes pancreatic cancer. Cancer Cell. (2018) 33:75–90.e7. doi: 10.1016/j.ccell.2017.11.007 29249692 PMC5760435

[45] Rosenne E Sorski L Shaashua L Neeman E Matzner P Levi B. *In vivo* suppression of NK cell cytotoxicity by stress and surgery: glucocorticoids have a minor role compared to catecholamines and prostaglandins. Brain Behav Immun. (2014) 37:207–19. doi: 10.1016/j.bbi.2013.12.007 PMC4322769 24333572

[46] Haldar R Ricon-Becker I Radin A Gutman M Cole SW Zmora O. Perioperative COX2 and β-adrenergic blockade improves biomarkers of tumor metastasis, immunity, and inflammation in colorectal cancer: A randomized controlled trial. Cancer. (2020) 126:3991–4001. doi: 10.1002/cncr.32950 32533792

[47] Wu WK Sung JJ Lee CW Yu J Cho CH. Cyclooxygenase-2 in tumorigenesis of gastrointestinal cancers: an update on the molecular mechanisms. Cancer Lett. (2010) 295:7–16. doi: 10.1016/j.canlet.2010.03.015 20381235

[48] Thaker PH Han LY Kamat AA Arevalo JM Takahashi R Lu C. Chronic stress promotes tumor growth and angiogenesis in a mouse model of ovarian carcinoma. Nat Med. (2006) 12:939–44. doi: 10.1038/nm1447 16862152

[49] Sood AK Armaiz-Pena GN Halder J Nick AM Stone RL Hu W. Adrenergic modulation of focal adhesion kinase protects human ovarian cancer cells from anoikis. J Clin Invest. (2010) 120:1515–23. doi: 10.1172/jci40802 PMC2860925 20389021

[50] Yang EV Kim SJ Donovan EL Chen M Gross AC Webster Marketon JI. Norepinephrine upregulates VEGF, IL-8, and IL-6 expression in human melanoma tumor cell lines: implications for stress-related enhancement of tumor progression. Brain Behav Immun. (2009) 23:267–75. doi: 10.1016/j.bbi.2008.10.005 PMC2652747 18996182

[51] Shakhar G Ben-Eliyahu S. Potential prophylactic measures against postoperative immunosuppression: could they reduce recurrence rates in oncological patients? Ann Surg Oncol. (2003) 10:972–92. doi: 10.1245/aso.2003.02.007 14527919

[52] Bartal I Melamed R Greenfeld K Atzil S Glasner A Domankevich V. Immune perturbations in patients along the perioperative period: alterations in cell surface markers and leukocyte subtypes before and after surgery. Brain Behav Immun. (2010) 24:376–86. doi: 10.1016/j.bbi.2009.02.010 19254757

[53] Inbar S Neeman E Avraham R Benish M Rosenne E Ben-Eliyahu S. Do stress responses promote leukemia progression? An animal study suggesting a role for epinephrine and prostaglandin-E2 through reduced NK activity. PloS One. (2011) 6:e19246. doi: 10.1371/journal.pone.0019246 21559428 PMC3084788

[54] Glasner A Avraham R Rosenne E Benish M Zmora O Shemer S. Improving survival rates in two models of spontaneous postoperative metastasis in mice by combined administration of a beta-adrenergic antagonist and a cyclooxygenase-2 inhibitor. J Immunol. (2010) 184:2449–57. doi: 10.4049/jimmunol.0903301 20124103

[55] Hashemi Goradel N Najafi M Salehi E Farhood B Mortezaee K. Cyclooxygenase-2 in cancer: A review. J Cell Physiol. (2019) 234:5683–99. doi: 10.1002/jcp.27411 30341914

[56] Hondermarck H Jobling P. The sympathetic nervous system drives tumor angiogenesis. Trends Cancer. (2018) 4:93–4. doi: 10.1016/j.trecan.2017.11.008 29458965

[57] Chaudry IH Ayala A Ertel W Stephan RN. Hemorrhage and resuscitation: immunological aspects. Am J Physiol. (1990) 259:R663–78. doi: 10.1152/ajpregu.1990.259.4.R663 2145776

[58] Reader J Holt D Fulton A. Prostaglandin E2 EP receptors as therapeutic targets in breast cancer. Cancer Metastasis Rev. (2011) 30:449–63. doi: 10.1007/s10555-011-9303-2 PMC3640271 22002714

[59] Brecht K Weigert A Hu J Popp R Fisslthaler B Korff T. Macrophages programmed by apoptotic cells promote angiogenesis via prostaglandin E2. FASEB J. (2011) 25:2408–17. doi: 10.1096/fj.10-179473 21450910

[60] van der Bij GJ Oosterling SJ Beelen RH Meijer S Coffey JC van Egmond M. The perioperative period is an underutilized window of therapeutic opportunity in patients with colorectal cancer. Ann Surg. (2009) 249:727–34. doi: 10.1097/SLA.0b013e3181a3ddbd 19387333

[61] Migita K Matsumoto S Wakatsuki K Kunishige T Nakade H Miyao S. Postoperative serum C-reactive protein level predicts long-term outcomes in stage I gastric cancer. J Surg Res. (2019) 242:323–31. doi: 10.1016/j.jss.2019.04.075 31129241

[62] Peiseler M Kubes P. More friend than foe: the emerging role of neutrophils in tissue repair. J Clin Invest. (2019) 129:2629–39. doi: 10.1172/jci124616 PMC6597202 31205028

[63] Chen Q Zhang L Li X Zhuo W. Neutrophil extracellular traps in tumor metastasis: pathological functions and clinical applications. Cancers (Basel). (2021) 13. doi: 10.3390/cancers13112832 PMC8200981 34204148

[64] Albrengues J Shields MA Ng D Park CG Ambrico A Poindexter ME. Neutrophil extracellular traps produced during inflammation awaken dormant cancer cells in mice. Science. (2018) 361. doi: 10.1126/science.aao4227 PMC6777850 30262472

[65] Kalogeris T Baines CP Krenz M Korthuis RJ. Ischemia/reperfusion. Compr Physiol. (2016) 7:113–70. doi: 10.1002/cphy.c160006 PMC5648017 28135002

[66] Selders GS Fetz AE Radic MZ Bowlin GL. An overview of the role of neutrophils in innate immunity, inflammation and host-biomaterial integration. Regener Biomater. (2017) 4:55–68. doi: 10.1093/rb/rbw041 PMC5274707 28149530

[67] Zappalà G McDonald PG Cole SW. Tumor dormancy and the neuroendocrine system: an undisclosed connection? Cancer Metastasis Rev. (2013) 32:189–200. doi: 10.1007/s10555-012-9400-x 23090259

[68] Erpenbeck L Schön MP. Neutrophil extracellular traps: protagonists of cancer progression? Oncogene. (2017) 36:2483–90. doi: 10.1038/onc.2016.406 27941879

[69] Derynck R Turley SJ Akhurst RJ. TGFβ biology in cancer progression and immunotherapy. Nat Rev Clin Oncol. (2021) 18(1):9–34. doi: 10.1038/s41571-020-0403-1 PMC9721352 32710082

[70] Kessenbrock K Plaks V Werb Z. Matrix metalloproteinases: regulators of the tumor microenvironment. Cell. (2010) 141:52–67. doi: 10.1016/j.cell.2010.03.015 20371345 PMC2862057

[71] Batlle E Massagué J. Transforming growth factor-β Signaling in immunity and cancer. Immunity. (2019) 50:924–40. doi: 10.1016/j.immuni.2019.03.024 PMC7507121 30995507

[72] Lamkin DM Srivastava S Bradshaw KP Betz JE Muy KB Wiese AM. C/EBPβ regulates the M2 transcriptome in β-adrenergic-stimulated macrophages. Brain Behav Immun. (2019) 80:839–48. doi: 10.1016/j.bbi.2019.05.034 PMC6660400 31132458

[73] Predina J Eruslanov E Judy B Kapoor V Cheng G Wang LC. Changes in the local tumor microenvironment in recurrent cancers may explain the failure of vaccines after surgery. Proc Natl Acad Sci U.S.A. (2013) 110:E415–24. doi: 10.1073/pnas.1211850110 PMC3562776 23271806

[74] Muliaditan T Caron J Okesola M Opzoomer JW Kosti P Georgouli M. Macrophages are exploited from an innate wound healing response to facilitate cancer metastasis. Nat Commun. (2018) 9:2951. doi: 10.1038/s41467-018-05346-7 30054470 PMC6063977

[75] Tham M Khoo K Yeo KP Kato M Prevost-Blondel A Angeli V. Macrophage depletion reduces postsurgical tumor recurrence and metastatic growth in a spontaneous murine model of melanoma. Oncotarget. (2015) 6:22857–68. doi: 10.18632/oncotarget.3127 PMC4673204 25762633

[76] Zhu H Leiss L Yang N Rygh CB Mitra SS Cheshier SH. Surgical debulking promotes recruitment of macrophages and triggers glioblastoma phagocytosis in combination with CD47 blocking immunotherapy. Oncotarget. (2017) 8:12145–57. doi: 10.18632/oncotarget.14553 PMC5355332 28076333

[77] Dedej T Lamaj E Marku N Ostreni V Bilali S. Alterations in homeostasis after open surgery. A prospective randomized study. G Chir. (2013) 34:202–9. doi: 10.11138/gchir/2013.34.7.202 PMC3915605 24091175

[78] Mafune K Tanaka Y. Influence of multimodality therapy on the cellular immunity of patients with esophageal cancer. Ann Surg Oncol. (2000) 7:609–16. doi: 10.1007/bf02725341 11005560

[79] Angka L Martel AB Kilgour M Jeong A Sadiq M de Souza CT. Natural killer cell IFNγ Secretion is profoundly suppressed following colorectal cancer surgery. Ann Surg Oncol. (2018) 25:3747–54. doi: 10.1245/s10434-018-6691-3 30187278

[80] Kim EY Hong TH. Changes in natural killer cell activity after surgery and predictors of its recovery-failure. J Surg Oncol. (2021) 124:1561–8. doi: 10.1002/jso.26636 34351633

[81] Angka L Khan ST Kilgour MK Xu R Kennedy MA Auer RC. Dysfunctional natural killer cells in the aftermath of cancer surgery. Int J Mol Sci. (2017) 18. doi: 10.3390/ijms18081787 PMC5578175 28817109

[82] Market M Baxter KE Angka L Kennedy MA Auer RC. The potential for cancer immunotherapy in targeting surgery-induced natural killer cell dysfunction. Cancers (Basel). (2018) 11. doi: 10.3390/cancers11010002 PMC6356325 30577463

[83] Pollock RE Lotzová E Stanford SD. Mechanism of surgical stress impairment of human perioperative natural killer cell cytotoxicity. Arch Surg. (1991) 126:338–42. doi: 10.1001/archsurg.1991.01410270082013 1825598

[84] Tai LH de Souza CT Bélanger S Ly L Alkayyal AA Zhang J. Preventing postoperative metastatic disease by inhibiting surgery-induced dysfunction in natural killer cells. Cancer Res. (2013) 73:97–107. doi: 10.1158/0008-5472.Can-12-1993 23090117

[85] Tai LH Zhang J Scott KJ de Souza CT Alkayyal AA Ananth AA. Perioperative influenza vaccination reduces postoperative metastatic disease by reversing surgery-induced dysfunction in natural killer cells. Clin Cancer Res. (2013) 19:5104–15. doi: 10.1158/1078-0432.Ccr-13-0246 23881927

[86] Reinhardt R Pohlmann S Kleinertz H Hepner-Schefczyk M Paul A Flohé SB. Invasive Surgery Impairs the Regulatory Function of Human CD56 bright Natural Killer Cells in Response to Staphylococcus aureus. Suppression of Interferon-γ Synthesis. PloS One. (2015) 10:e0130155. doi: 10.1371/journal.pone.0130155 26090673 PMC4474941

[87] Benish M Bartal I Goldfarb Y Levi B Avraham R Raz A. Perioperative use of beta-blockers and COX-2 inhibitors may improve immune competence and reduce the risk of tumor metastasis. Ann Surg Oncol. (2008) 15:2042–52. doi: 10.1245/s10434-008-9890-5 PMC3872002 18398660

[88] Goldfarb Y Sorski L Benish M Levi B Melamed R Ben-Eliyahu S. Improving postoperative immune status and resistance to cancer metastasis: a combined perioperative approach of immunostimulation and prevention of excessive surgical stress responses. Ann Surg. (2011) 253:798–810. doi: 10.1097/SLA.0b013e318211d7b5 21475023

[89] Seth R Tai LH Falls T de Souza CT Bell JC Carrier M. Surgical stress promotes the development of cancer metastases by a coagulation-dependent mechanism involving natural killer cells in a murine model. Ann Surg. (2013) 258:158–68. doi: 10.1097/SLA.0b013e31826fcbdb 23108132

[90] Pollock RE Lotzová E Stanford SD. Surgical stress impairs natural killer cell programming of tumor for lysis in patients with sarcomas and other solid tumors. Cancer. (1992) 70:2192–202. doi: 10.1002/1097-0142(19921015)70:8<2192::aid-cncr2820700830>3.0.co;2-6 1394051

[91] Iannone F Porzia A Peruzzi G Birarelli P Milana B Sacco L. Effect of surgery on pancreatic tumor-dependent lymphocyte asset: modulation of natural killer cell frequency and cytotoxic function. Pancreas. (2015) 44:386–93. doi: 10.1097/mpa.0000000000000288 PMC4358707 25621568

[92] Velásquez JF Ramírez MF Ai DI Lewis V Cata JP. Impaired immune function in patients undergoing surgery for bone cancer. Anticancer Res. (2015) 35:5461–6. 26408709

[93] Palumbo JS Talmage KE Massari JV La Jeunesse CM Flick MJ Kombrinck KW. Platelets and fibrin(ogen) increase metastatic potential by impeding natural killer cell-mediated elimination of tumor cells. Blood. (2005) 105:178–85. doi: 10.1182/blood-2004-06-2272 15367435

[94] Krall JA Reinhardt F Mercury OA Pattabiraman DR Brooks MW Dougan M. The systemic response to surgery triggers the outgrowth of distant immune-controlled tumors in mouse models of dormancy. Sci Transl Med. (2018) 10. doi: 10.1126/scitranslmed.aan3464 PMC6364295 29643230

[95] Tang F Tie Y Tu C Wei X. Surgical trauma-induced immunosuppression in cancer: Recent advances and the potential therapies. Clin Transl Med. (2020) 10:199–223. doi: 10.1002/ctm2.24 32508035 PMC7240866

[96] Phillips JD Knab LM Blatner NR Haghi L DeCamp MM Meyerson SL. Preferential expansion of pro-inflammatory Tregs in human non-small cell lung cancer. Cancer Immunol Immunother. (2015) 64:1185–91. doi: 10.1007/s00262-015-1725-1 PMC4891203 26047578

[97] Saito Y Shimada M Utsunomiya T Morine Y Imura S Ikemoto T. Regulatory T cells in the blood: a new marker of surgical stress. Surg Today. (2013) 43:608–12. doi: 10.1007/s00595-013-0517-5 23412515

[98] Wang B Zhang Z Liu W Tan B. Targeting regulatory T cells in gastric cancer: Pathogenesis, immunotherapy, and prognosis. BioMed Pharmacother. (2023) 158:114180. doi: 10.1016/j.biopha.2022.114180 36586241

[99] Wang Y Ding Y Guo N Wang S. MDSCs: key criminals of tumor pre-metastatic niche formation. Front Immunol. (2019) 10:172. doi: 10.3389/fimmu.2019.00172 30792719 PMC6374299

[100] Veglia F Sanseviero E Gabrilovich DI. Myeloid-derived suppressor cells in the era of increasing myeloid cell diversity. Nat Rev Immunol. (2021) 21:485–98. doi: 10.1038/s41577-020-00490-y PMC7849958 33526920

[101] Gabrilovich DI Nagaraj S. Myeloid-derived suppressor cells as regulators of the immune system. Nat Rev Immunol. (2009) 9:162–74. doi: 10.1038/nri2506 PMC2828349 19197294

[102] Lu Z Zou J Li S Topper MJ Tao Y Zhang H. Epigenetic therapy inhibits metastases by disrupting premetastatic niches. Nature. (2020) 579:284–90. doi: 10.1038/s41586-020-2054-x PMC8765085 32103175

[103] Wang J Yang L Yu L Wang YY Chen R Qian J. Surgery-induced monocytic myeloid-derived suppressor cells expand regulatory T cells in lung cancer. Oncotarget. (2017) 8:17050–8. doi: 10.18632/oncotarget.14991 PMC5370021 28178645

[104] Pan Y Yu Y Wang X Zhang T. Tumor-associated macrophages in tumor immunity. Front Immunol. (2020) 11:583084. doi: 10.3389/fimmu.2020.583084 33365025 PMC7751482

[105] Arai Y Saito H Ikeguchi M. Upregulation of TIM-3 and PD-1 on CD4+ and CD8+ T cells associated with dysfunction of cell-mediated immunity after colorectal cancer operation. Yonago Acta Med. (2012) 55:1–9. 24031134 PMC3727487

[106] Xu P Zhang P Sun Z Wang Y Chen J Miao C. Surgical trauma induces postoperative T-cell dysfunction in lung cancer patients through the programmed death-1 pathway. Cancer Immunol Immunother. (2015) 64:1383–92. doi: 10.1007/s00262-015-1740-2 PMC11028497 26183035

[107] Sun Z Mao A Wang Y Zhao Y Chen J Xu P. Treatment with anti-programmed cell death 1 (PD-1) antibody restored postoperative CD8+ T cell dysfunction by surgical stress. BioMed Pharmacother. (2017) 89:1235–41. doi: 10.1016/j.biopha.2017.03.014 28320090

[108] Leibner E Andreae M Galvagno SM Scalea T. Damage control resuscitation. Clin Exp Emerg Med. (2020) 7:5–13. doi: 10.15441/ceem.19.089 32252128 PMC7141982

[109] van der Pas MH Haglind E Cuesta MA Fürst A Lacy AM Hop WC. Laparoscopic versus open surgery for rectal cancer (COLOR II): short-term outcomes of a randomised, phase 3 trial. Lancet Oncol. (2013) 14:210–8. doi: 10.1016/s1470-2045(13)70016-0 23395398

[110] Torres A Torres K Paszkowski T Staśkiewicz GJ Maciejewski R. Cytokine response in the postoperative period after surgical treatment of benign adnexal masses: comparison between laparoscopy and laparotomy. Surg Endosc. (2007) 21:1841–8. doi: 10.1007/s00464-007-9260-6 17356933

[111] Donmez T Uzman S Yildirim D Hut A Avaroglu HI Erdem DA. Is there any effect of pneumoperitoneum pressure on coagulation and fibrinolysis during laparoscopic cholecystectomy? PeerJ. (2016) 4:e2375. doi: 10.7717/peerj.2375 27651988 PMC5018660

[112] Carless PA Henry DA Moxey AJ O’Connell D Brown T Fergusson DA. Cell salvage for minimising perioperative allogeneic blood transfusion. Cochrane Database Syst Rev. (2010) 2010:Cd001888. doi: 10.1002/14651858.CD001888.pub4 17054147

[113] Waters JH Yazer M Chen YF Kloke J. Blood salvage and cancer surgery: a meta-analysis of available studies. Transfusion. (2012) 52:2167–73. doi: 10.1111/j.1537-2995.2011.03555.x 22321196

[114] Oosterling SJ van der Bij GJ Mels AK Beelen RH Meijer S van Egmond M. Perioperative IFN-alpha to avoid surgically induced immune suppression in colorectal cancer patients. Histol Histopathol. (2006) 21:753–60. doi: 10.14670/hh-21.753 16598674

[115] Francois B Jeannet R Daix T Walton AH Shotwell MS Unsinger J. Interleukin-7 restores lymphocytes in septic shock: the IRIS-7 randomized clinical trial. JCI Insight. (2018) 3. doi: 10.1172/jci.insight.98960 PMC5922293 29515037

[116] Shi LZ Fu T Guan B Chen J Blando JM Allison JP. Interdependent IL-7 and IFN-γ signalling in T-cell controls tumour eradication by combined α-CTLA-4+α-PD-1 therapy. Nat Commun. (2016) 7:12335. doi: 10.1038/ncomms12335 27498556 PMC4979067

[117] Matzner P Sorski L Haldar R Shaashua L Benbenishty A Lavon H. Deleterious synergistic effects of distress and surgery on cancer metastasis: Abolishment through an integrated perioperative immune-stimulating stress-inflammatory-reducing intervention. Brain Behav Immun. (2019) 80:170–8. doi: 10.1016/j.bbi.2019.03.005 30851377

[118] Krieg AM. Therapeutic potential of Toll-like receptor 9 activation. Nat Rev Drug Discovery. (2006) 5:471–84. doi: 10.1038/nrd2059 16763660

[119] Goldfarb Y Benish M Rosenne E Melamed R Levi B Glasner A. CpG-C oligodeoxynucleotides limit the deleterious effects of beta-adrenoceptor stimulation on NK cytotoxicity and metastatic dissemination. J Immunother. (2009) 32:280–91. doi: 10.1097/CJI.0b013e31819a2982 PMC2738855 19242372

[120] Darvin P Toor SM Sasidharan Nair V Elkord E. Immune checkpoint inhibitors: recent progress and potential biomarkers. Exp Mol Med. (2018) 50:1–11. doi: 10.1038/s12276-018-0191-1 PMC6292890 30546008

